# The investigation of diesel soot emission using instrument combination of multi-wavelength photoacoustic spectroscopy and scanning mobility particle sizer

**DOI:** 10.1038/s41598-024-52832-8

**Published:** 2024-01-26

**Authors:** Tibor Ajtai, Noémi Utry, Máté Pintér, Abdul Rahman, Boldizsár Kurilla, Gábor Sárossy, László Deák, Jenő Baladincz, Péter Raffai, Gábor Szabó, Zoltán Bozóki

**Affiliations:** 1 Department of Optics and Quantum Electronics, University of Szeged, 9. Dóm Square, Szeged, 6720 Hungary; 2 HUN-REN-SZTE Research Group for Photoacoustic Monitoring of Environmental Processes, Dóm ter 9, Szeged, 6720 Hungary; 3 MOL Ltd. Duna Refinery, Százhalombatta, 2440 Hungary

**Keywords:** Environmental impact, Energy science and technology, Nanoscience and technology

## Abstract

The parallel measurements of wavelength dependent optical absorption, particle number size distribution have made by a multi wavelength photoacoustic spectrometer (4λ-PAS) and scanning mobility particle sizer (SMPS) respectively at different modes of a diesel engine using two different types of fuel. The thermal evolution of the emission was also investigated using posterior temperature treatment of emission. The bimodal size distribution of emitted particles at a set reference temperature has been observed regardless of the applied fuel at idle. However, the emitted particulate assembly had lognormal size distribution falls into the accumulation mode at all other defined engine modes and both fuel types. The total number- and volume concentration (TNC and TVC) showed retrograde tendency with the increasing torque and rpm independently of the applied fuel types. The TNC values decreased up to 50% for both fuels with engine operation changes from idle engine mode(em#1) to low engine mode(em#2). With further increase in torque and rpm of engine, the change in TNC is negligible. On the other hand, the TVC remains more or less the same for idle to low engine mode transition and increased more than 60% for high mode (em#3) transition. The Optical Absorption Coefficient (OAC) values measured at the operational wavelengths of the 4λ-PAS instrument decreased at all wavelengths with increasing rpm and torque. The wavelength dependency quantified by Aerosol Ängström Exponent (AAE) was applied here for qualitative analysis of the carbonaceous emission and showed decreased values towards the higher engine speed and torque output of the engine. The proposed technique can be used as real-time, precise and accurate measurement of light absorption by DPM aerosols, which opens up novel possibilities for the volatility and thermal evolution investigation of diesel emissions.

## Introduction

The investigation of Diesel Particulate Matter (DPM) is subject to continuously increasing scientific interest due to its adverse health and climate impact. Diesel emitted aerosols are one of the most prominent sources of Light Absorbing Carbonaceous (LAC) particulate matter. The LAC is one of the most harmful air pollutants^1^ and as the major fraction of Black Carbon (BC), the second most important climate relevant atmospheric constituent^2^, due to its small size combined with high number-concentration, high surface area per unit volume and its adsorption ability to toxic substances. Despite of its importance, both climatic and health effects of DPM are quite uncertain, therefore diesel engine emissions are under scrutiny in various aspects^3^. In this regard the further understanding of its fundamentals, the better characterisation of DPM regarding methodology/instrumentation and reduction of tailpipe emissions in engineering, are all important and latest scientific issues. Emission based fuel development is the promising alternative for the reduction of emission and also for eco-friendly fuel development. However, it requires the precise measurement of the exhausted particles dispersed in a reactive and turbulent gas matrix, which poses a range of challenges for instrument and methodology developers. The DPM is a highly versatile and complex mixture of volatile organic and non-volatile inorganic substances with diverse physicochemical properties. During the particle evolution process, the volatile to non-volatile ratio of the DPM is dynamically changing with temperature. The volatility of diesel emission is the indicator of the organic carbon content of particulate which evaporates at a given temperature. Vapour species with high molecular weight can condense on the non-volatile soot by heterogeneous- or form new individual particles by homogeneous condensations. These processes affect the chemical composition and also the mixing states of DPM. The measured physicochemical properties of DPM are strongly dependent on the type and operational condition of the engine as well as fuel compositions^4^. The regulated parameters of DPM are the number and mass concentrations. However, in describing DPM's air quality and climate relevance their usefulness is limited. For effective emission based fuel development, the complete characterisation of the exhausted DPM is deemed essential. This requires real time or preferably in-situ methodologies adopted to rigid measurement conditions in a highly turbulent and reactive ambient gas. The size distribution, volatility classification and spectral response of diesel soot are critical parameters in many aspects. A powerful method for volatility classification of DPM is based on measuring the size distribution of the temperature treated and denuded aerosol was demonstrated earlier^5^. Using a thermodenuder (TD) for pre-treating the exhausted DPM makes the volatility classification of DPM possible and also provides an indirect opportunity to investigate the particle states associated with a particular temperature in the exhaust pipe under steady state measurement conditions.

The LAC is an atmospheric composite having positive radiative forcing with the highest uncertainty in calculations^6^. Therefore, aerosol light absorption is also a crucial climate relevant quantity. The absorption spectra of LAC is quantified by its wavelength dependency. The AAE – the slope of the absorption spectra in log–log scale—is the only real-time measurable physical quantity with composition and air quality relevancies^7,8^. Hence, the precise and accurate measurement of optical absorption and its wavelength dependence is important for characterisation of DPM in the context of its environmental impact. The light absorption of aerosol is one of the most difficult quantities to measure^9^. The most widespread methods for absorption measurement are based on the transmission measurement of filter-accumulated aerosol^10,11^, however it suffers from various methodological and analytical artefacts because of filter usage^12^. Aerosol researchers are in the common platform that photoacoustic spectroscopy (PAS) is the most suitable method for in-situ (filter free), precise and accurate measurement of light absorption by aerosols^13^. The PAS is a direct, fast and easily calibrated method, which does not contain the fundamental difficulties associated with filter based measurements or in-situ measurement using the difference of extinction and scattering^9^. Although the favourable attributes of PAS against alternatives have been well known for a long while, the photoacoustic technique has recently been developed into a stable method and is now becoming an accepted standard for measuring aerosol light absorption even for diesel exhaust measurements ^14^. In the last decade, the appearance of multi-wavelength PA instruments opened up novel opportunity for spectral characterisation of atmospheric LAC^7,8,15^. Due to its complex air quality and climate relevancies, the parallel measurement of size distribution and the spectral responses of thermally pre-treated DPM provides a powerful opportunity to better understand its environmental impact and serve as a novel method for emission based fuel development.

There are some studies that characterize the size distribution and varying elemental carbon content in DPM depending on the engine type and operating points^16–18^. Despites of its feasibility for the accurate determination of spectral response of DPM, there is only one study that has been reported for application of multi-wavelength PAS for exhaust particles of diesel engine at constant speed modes using commercial fuel type^19^. The current article demonstrates the applicability of instrument combination of 4λ-PAS and SMPS for the parallel measurement of wavelength dependent optical absorption and size distribution of the diesel emission using different type of fuels. This is the first demonstrative study which describes the effect of biomass content of fuel on the wavelength dependent absorption quantified by AAE at different engine modes (specified by rpm and torque) in the context of its thermal stability. The presented approach opens up novel possibility to deeper understand the emission characteristic of diesel soot. We present the experimental results of number concentration, size distribution and absorption spectra measurements of DPM as function of the operational conditions of diesel engines using different fuel types by PA-TD and SMPS. The presented results give more accurate and dynamic spectral response of DPM in comparison to the previous studies. We also investigate the measured quantities as the function of thermal stability.

## Material and methods

**Figure 1:**
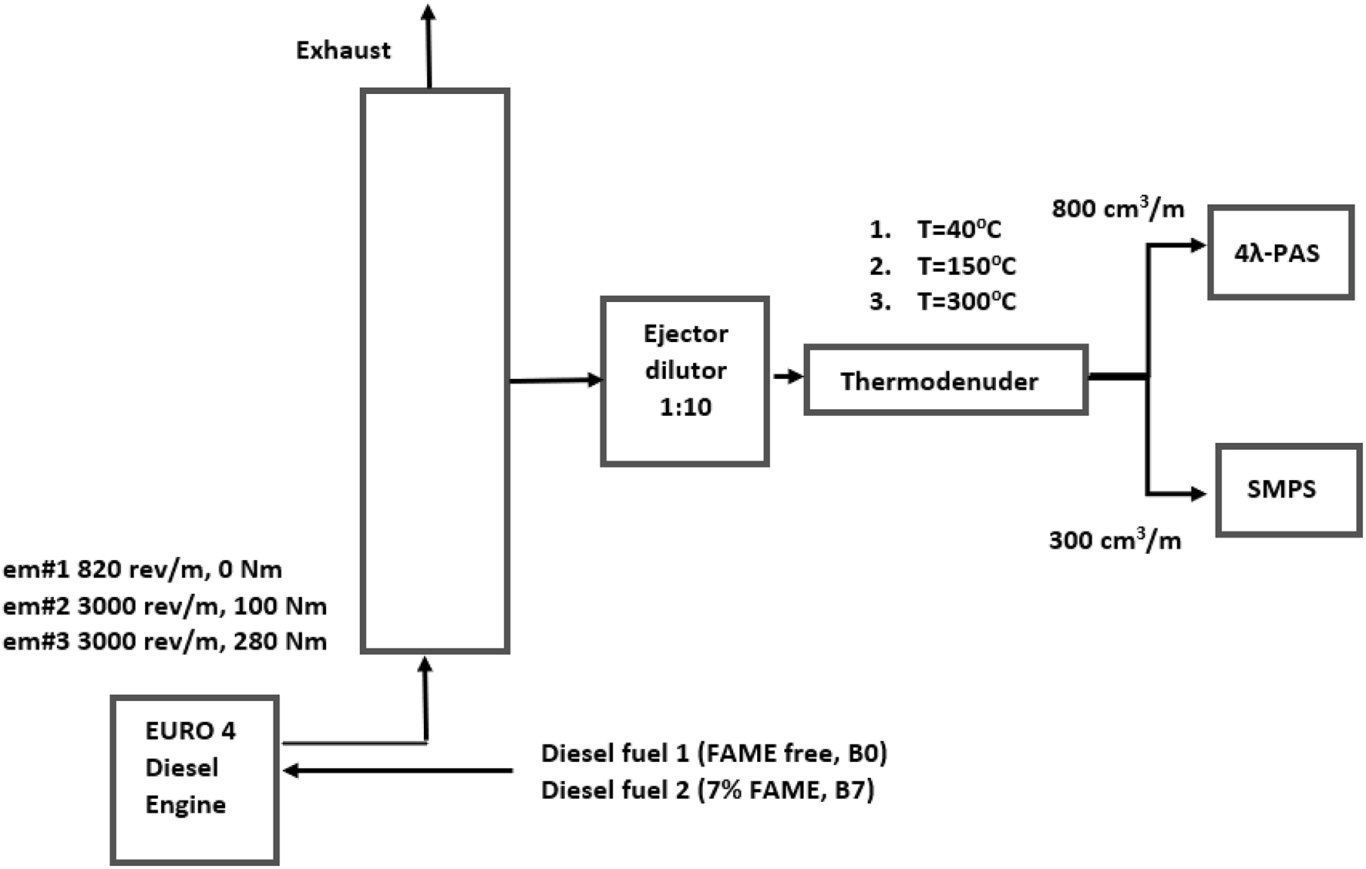
The experimental set-up of the sampling system. Operatory conditions of engine, type of fuels, posterior temperature treatment conditions.

The experimental setup for the measurement of size distribution and absorption responses of DPM is shown in Fig. 1.

The measurements were carried out in the engine test laboratory of Refining Research and Innovation MOL Experiment Centre at Százhalombatta, Hungary. A four cylinder diesel engine (2 L, turbo charged, EURO4 PC, common rail injection system) was used to generate DPM. Exhaust gas was diluted by a factor of 10 using an ejector diluter in an isokinetic way (PALAS GmbH VKL10). The characteristics performances of VKL10 are well defined by the manufacturer. At any operational conditions of the engine the exhausted particulate matter was thermally treated before measuring. For reference measurements, TD’s heating unit was set to 40 °C. For volatility measurements, the TD was heated up to 150 °C and 300 °C. A detailed description of the low-flow TD unit is described in detail elsewhere^20^. This modular type TD concept was developed for examination of exhaust-born particles having high volatile material content. Characteristic parameters of the TD applied here were defined and optimised for the particular flow requirements of the applied instrumentation and minimizing the loss effects^21^. The number concentration and the size distribution of the temperature treated aerosol assembly were measured by SMPS (SMPS, Grimm Aerosol Technique, Germany), consisting of two parts. The sampled aerosol particle assembly is first lead through a Long Differential Mobility Analyser (LDMA, Model#5.500), in which particle classification based on their electrical mobility takes place. Then the size-selected particles are counted by a Condensation Particle Counter (CPC, Modell#5.411). The sheet and aerosol flow rates of the SMPS were set to 3 and 0.3 L/minute respectively. In this set, the SMPS measure the size distribution from 11.1 to 1083.3 nm with ± 0.5 nm resolution at 11.1 nm and ± 30 nm at 1083.3 nm. To eliminate the shielding artefact coincidence correction on the measured data was applied. The multiple charge correction factors were employed to the operation of SMPS during whole campaign. The optical absorption coefficient and its wavelength dependency were measured by our 4λ-PAS^22^. The operation principles and the characteristic parameters of 4λ-PAS are described in detail elsewhere. Briefly, when the power modulated light is absorbed by LAC, the absorbed energy is released as heat resulting in a periodic pressure fluctuation. This pressure fluctuation is further amplified by a resonator, when the light source is modulated at its resonance frequency. The amplified pressure fluctuation is detected by a microphone and converted into an electrical signal, which is proportional with the absorption efficiency and the mass concentration of the measured LAC. An experimentally determined calibration factor ensures the correct deduction of the OAC from the measured electrical signal^22^. The 4λ-PAS measures the OAC at four different wavelengths (266 nm, 355 nm, 532 nm and 1064 nm) in separate but identical cells simultaneously. The flow rate of each cell is set to 0.3 L/minute. The instrument sensitivity is below 1 Mm^−1^ (inverse megameter) at 1064 nm, ~ 5 Mm^−1^ at 532 nm and ~ 23 Mm^−1^ at 355 nm and 35 Mm^−1^ at 266 nm, which corresponds to a mass sensitivity below or around 1 µg/m^3^ at all wavelengths. The instrument’s uncertainty was found to be 9%@266 nm, 11%@355 nm, 4%@532 nm and 22%@1064 nm^22,23^. The engine was powered by two type of commercially available fuels named B0 and B7 in this study (EN 590). The petroleum based B0 (biofree) fuel was used for reference. For the investigation of biofuel effect, B0 was blended with 7% FAME (Fatty Acid Methyl Ester) (B7). The engine was run to three distinct operational conditions during the measurements (Table 1). The PNSD and spectral response of DPM were measured at each operational mode of the engine using pure (B0) and FAME mixed diesel fuels (B7) at three different TD temperatures. The operational conditions of the engine, fuel types and sampling temperatures are summarised in Table 1. The characteristic parameters of the engine and the applied fuels are summarised in Tables 2 and 3.

**Table 1:** Fuel types, engine modes and sampling temperature.

#	Fuel Type
I	100% Diesel (B0)
II	7% FAME (B7)
#	Engine mode
1	0 Nm, 820 rpm
2	100 Nm, 3000 rpm
3	280 Nm, 3000 rpm
#	Thermodenuder temperature
a	40 °C
b	150 °C
c	300 °C

**Table 2:** Characteristic parameters of the test engine.

Bore	85 mm
Stroke	88 mm
Number of cylinders	4
Engine layout	inline
Compression ratio	16.7/1
Displacement	1997 cc
Injection	direct injection
Fuel supply system	common rail
Charging	turbo charged
Rated power	80 kW
Rated torque	400 nm

**Table 3:** Characteristic parameters of the applied fuels.

Fuel type	Density (kg/dm^3^) 15 °C	FAME content (% V/V)	Cetane number	Initial boiling point (°C)	Final boiling point (°C)
B0	0.84	< 0.05	51.3	175.6	367.7
B7	0.842	6.9	50.2	174.3	366.3

## Results and discussion

### Effect of the operational condition of engine, fuel type and temperature treatment on the measured size distribution function

**Figure 2:**
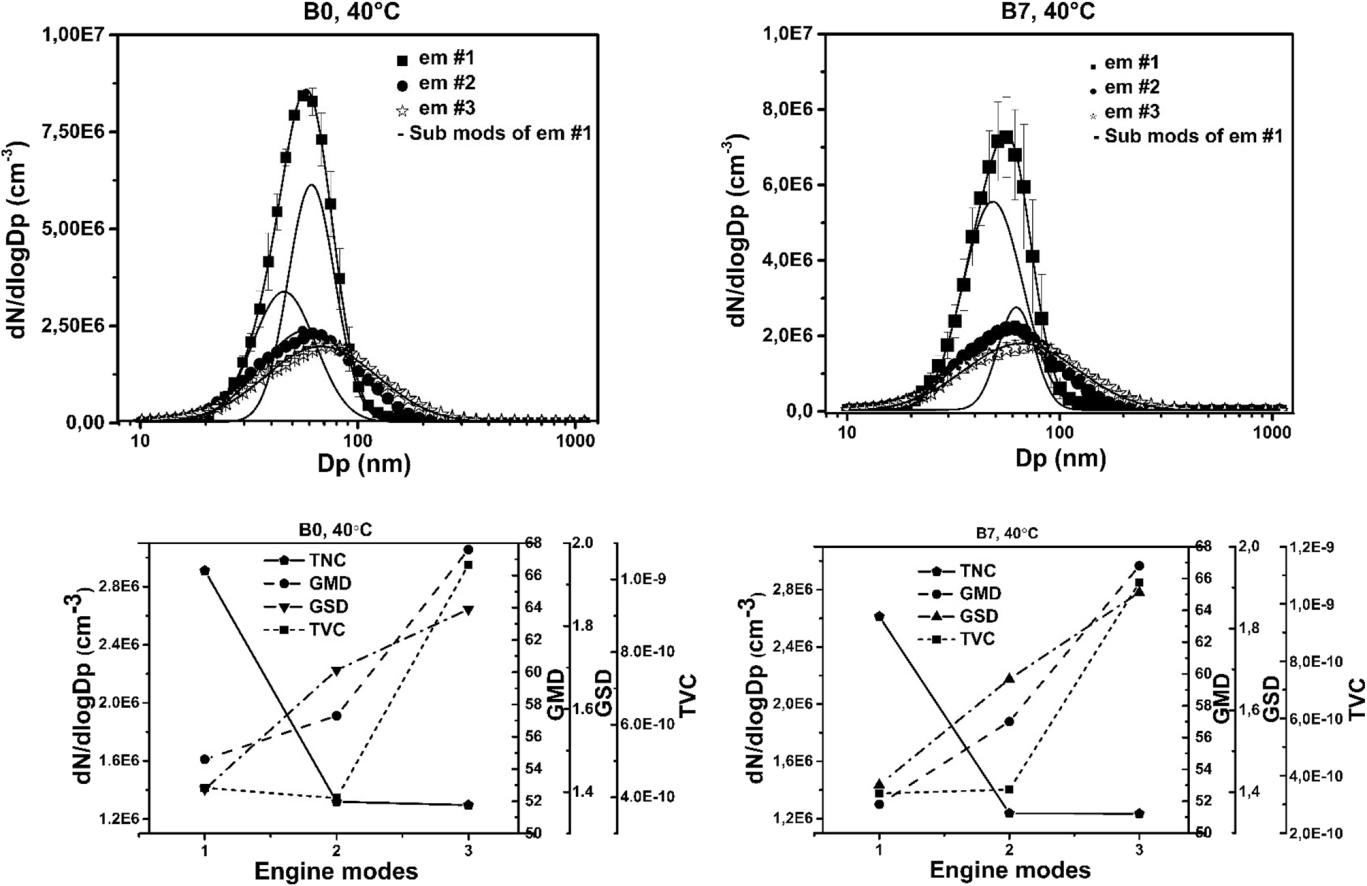
Size distributions and their characteristic parameters using B0 and B7 fuels at three different operatory parameters of the test engine. The TNC, GMD, GSD and TVC.

The results of number concentration and size distribution measurements using the fuels and engine setting (Tables 1 and 2) are shown in Fig. 2 and summarised in Table 4. The data was analysed using a lognormal fitting algorithm^24^. The characteristic parameters of the identified modes including geometric median diameter (GMD), geometric standard deviation (GSD) and the TNC were deduced from the fittings. The TVC was also inferred from the population of number concentration using a simple spherical approach as the function of the mobility diameter in our calculations.

**Table 4:** Characteristic parameters of the size distributions measured under each set of engine operation conditions and at three sampling temperatures using two types of fuels.

Fuel type and temperature	TNC (*10^6^) (cm^−3^)	GSD_N_ (nm)		GMD (nm)		TVC (*10^–10^) (cm^−3^)
Engine mode #1						
I.a	2.91	108.5	87	54.6	45.7	4.26
			88.1		60.2	
II.a	2.61	104.4	91	51.8	47.8	3.38
			88.7		61.6	
I.b	2.17	81.4		38.2		1.41
II.b	2.15	72.1		35.3		1.20
I.c	1.05	51.5		24.2		0.982
II.c	1.02	48.3		24		0.709
Engine mode #2						
I.a	1.32	165.6		57.3		3.98
II.a	1.24	160.8		57		3.52
I.b	1.09	166.8		55.1		3.15
II.b	0.98	171.9		55.5		2.98
I.c	0.98	163.4		54		2.59
II.c	0.91	169		55.2		2.66
Engine mode #3						
I.a	1.29	228.8		67.6		10.4
II.a	1.23	238.6		66.8		10.7
I.b	1.13	230.9		68.2		9.65
II.b	0.94	239.5		68.5		8.28
I.c	0.89	220.3		68		6.91
II.c	0.89	228.6		66.1		6.99

First, the size distribution was analysed at reference temperature (40 °C). At idle engine mode (em#1) in Table 1, we identified a bimodal size distribution regardless of the applied fuel type (Fig. 2). However, the coefficient of determination (R^2^) was found to be high in both cases even with mono-modal fitting (Table 4). Therefore, more detailed analysis of fitting deemed essential to reveal the structure of the modes using multiple-fitting algorithm with maximizing R^2^. A more detailed investigation of soot aerosols with multi-modal distributions would require extensive and specialized measurements which are beyond the scope of this study. Here we give a possible and plausible interpretation based on the general explanation of particle evolution in combustion processes. The sub-mode with the smaller GMD value includes particles consisting mainly of volatile organic compounds manifested in liquid phase, and developed through heterogeneous condensation, while the other mode is likely to be populated by soot nano-fractal aggregates having graphitic or turbostratic molecule structure in solid phase and evolved through nucleation, condensation and aggregation processes^5,25^. Of course, each type of aerosol can occur in both reverse sub-mode, but with limited concentrations. In Comparison with B0 and B7 fuel emissions at idle speed (em#1), change in emission strength of the sub-modes can be observed. Using B0 fuel the TNC of sub-mode with higher GMD value is dominant, while in case of B7, emission in the smaller sub-mode is more effective (Table 4). At (em#2 in Table 1) and (em #3 in Table 1) mono-modal size distribution can be observed (Fig. 2). In general, both fuels provide similar emission characteristics (i.e. GMD and GSD values) at (em#2) and (em#3), however, at any operational condition of the engine, the TNC has found to be even more than 10% smaller in case of B7 than in B0 fuel (Fig. 2 and Table 4). At (em#1) to (em#2) transition the TNC significantly decreased, while at the em#2—em#3 transition it remained nearly same (Fig. 2). However, it is noteworthy that in the latter transition, the TNC values and the population of particles show high degree of similarity but the ratio of smaller to lager particulates has changed inside the population. At higher torque (280Nm) the population contains fewer small (smaller than GMD), but more big particles (larger than GMD) compared to the lower torque (100Nm) case (Fig. 2). This seemingly marginal change in size distribution resulted in significant differences in TVC values. The more large particles tends to make the accumulation mode while more small size particles resides in the nucleation mode^26^. The B0 and B7 fuels exhibit different strengths in modes structure at idle engine mode. The TVC values substantially increased in the (em#2) to (em#3) transition (Fig. 2). Similarly, despite of the remarkable differences in TNC values measured in the (em#1) to (em#2) transition, the deviation in population statistics resulted in quite similar TVC values.

Lastly, the GSD and GMD values also show an increasing trend with increasing bias in the order of em’s respectively (Fig. 2).

Thermal behaviour of the emitted particulate matter is also analysed in this study. At higher temperatures, mono-modal size distribution has been observed independently of the applied fuel and the operational parameters of the engine (Table 4). With increasing temperature, the emission efficiency in number concentration (TNC), the characteristic size (GMD) and the width of the population (GSD) behave very alike at all measurement conditions. The TNC, GMD, and GSD decrease towards the higher temperatures. The changes in population characteristics resulted in a smaller number concentration and characteristic size (GMD) with increasing temperature can be explained as: the vapour-particle ratio of the exhaust is continuously in dynamic thermal equilibrium with its ambience. So, at higher temperatures the volatile molecules condensed on the non-volatile elemental carbon fractals at an earlier stage of particle evolution process, which evaporates at higher temperature. However, it is worth to note, that the particle restructuring is responsible for the change in population statistics since the organic content of the particle is evaporated, resulting in smaller characteristic size therefore the characteristic size of the particle assembly (GMD) decreases. Similarly, the volatile particulates evolved through condensation are partly or completely evaporated at higher temperatures, yields to decreasing TNC values^3^. The result of these two effects using higher temperature in TD unit manifested in shift in characteristic size towards the smaller particles and cause reduction in number-concentration too. Overall, the 150 °C to 300 °C transition shows further but moderate decrease in number concentration and changes in population statistics relative to the 40 °C to 150 °C transition regardless of fuel type and operational conditions of the engine (Table 4).

### Effect of the operational condition of engine, fuel type and temperature treatment on the measured absorption spectra

**Figure 3:**
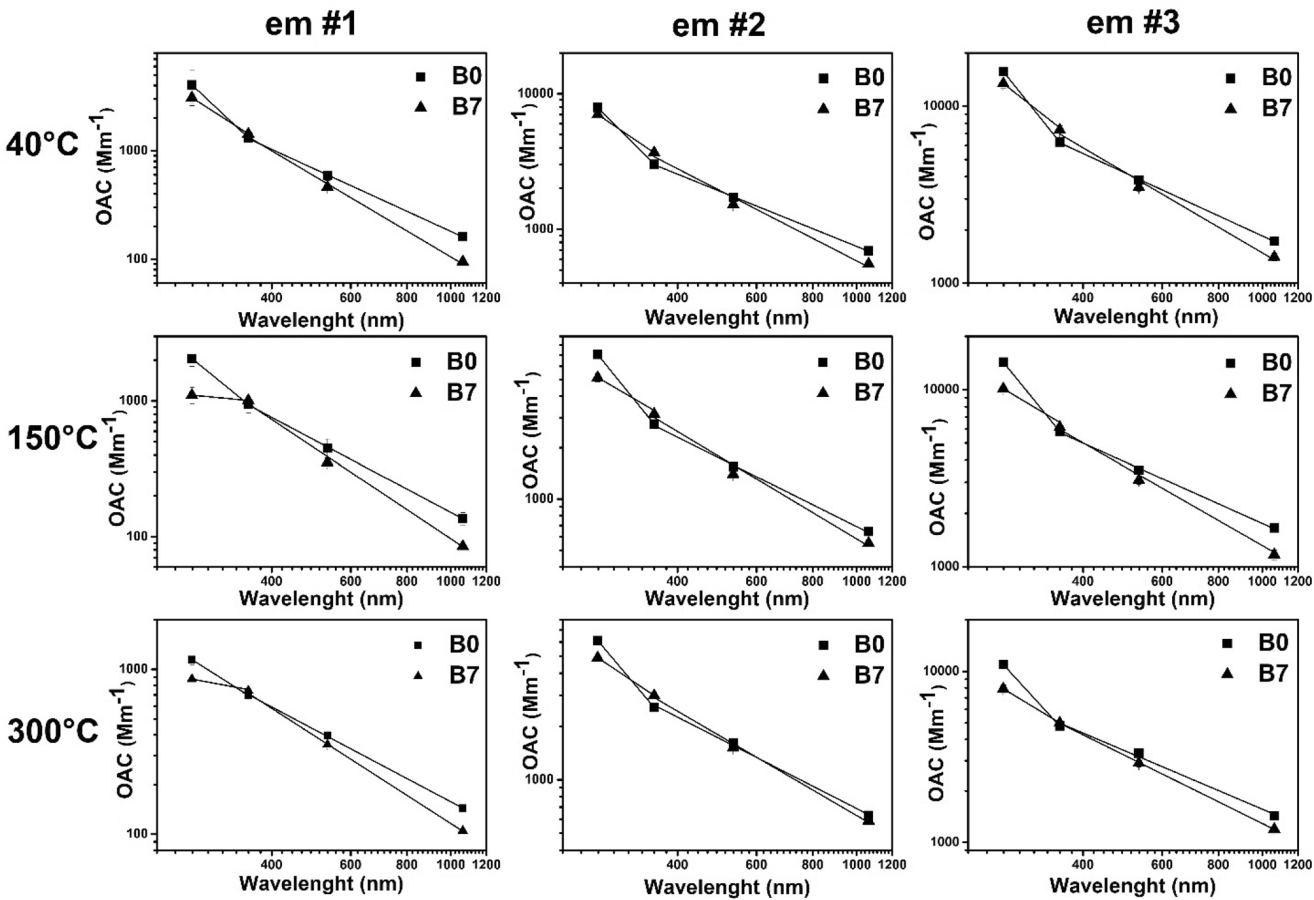
OAC measured at the wavelengths of the photoacoustic instruments at each measurement setting.

**Figure 4:**
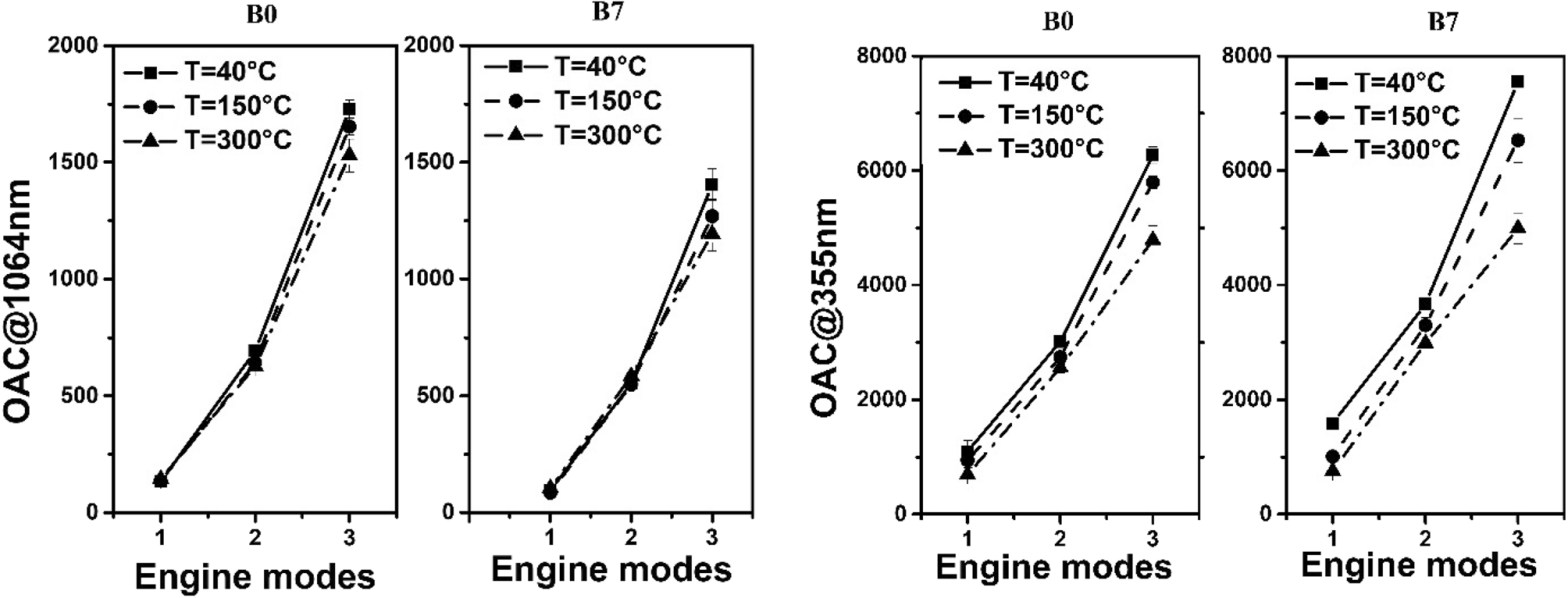
OAC measured at 1064 nm and 355 nm in the function of em’s at three different temperatures using two types of fuels.

**Figure 5:**
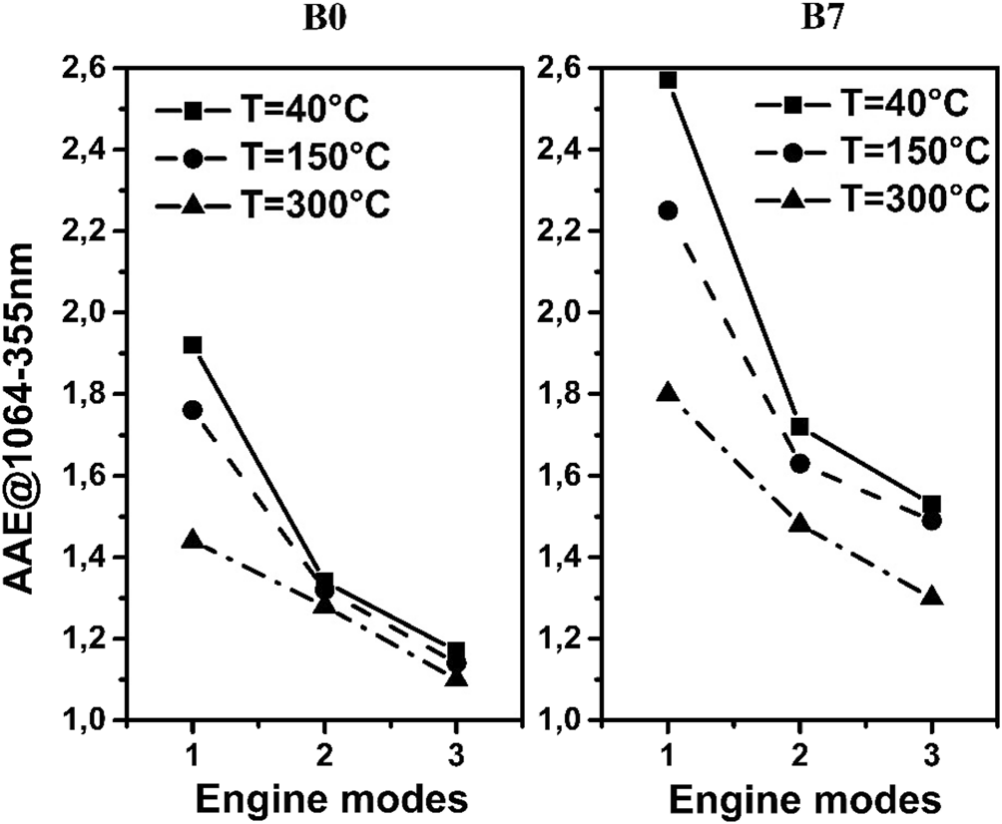
AAE of diesel emission in the function of engine modes using different type of fuels and sampling temperature.

The optical absorption at the operational wavelengths of the 4λ-PAS and its wavelength dependency quantified by the AAE measured at different engine operation conditions, fuel types and temperatures are shown in Figs. 3, 4, 5 and summarized in Table 5.

**Table 5:** The optical absorption coefficient and AAE values measured under each set of engine operation conditions and at three sampling temperatures using two types of fuels.

Wavelength (nm)	T = 40 °C		T = 150 °C		T = 300 °C	
	OAC Mm^−1^		OAC Mm^−1^		OAC Mm^−1^	
	B0	B7	B0	B7	B0	B7
*em#1*						
1064	160.6	94.9	135.9	84.9	143.7	104.8
532	591.6	461.8	448.4	353	398.5	309.5
355	1317.9	15,923	943.2	1008.3	695.6	754.1
266	4056.9	3085	2057.6	1102.5	1147.1	872.7
AAE@1064-355 nm	1.92	2.57	1.76	2.25	1.43	1.8
*em#2*						
1064	692.7	555.1	642.6	549.1	627.3	585.7
532	1715	1521.3	1542.4	1397.3	1615.4	1516.7
355	3018.1	3670.1	2746.1	3302.6	2562.5	2989.2
266	7903.6	7011.8	7031.3	5152.5	6106.8	4887.4
AAE@1064-355 nm	1.34	1.72	1.32	1.63	1.28	1.48
*em#3*						
1064	1728.3	1404.9	1652.8	1269.2	1429.9	1193.3
532	3815.3	3380.4	3520.5	2883.2	3316.6	2909.4
355	6266.3	7547.7	5794.3	6529.6	4786.4	4991.1
266	15,680.4	13,416.8	14,292.7	10,112.7	10,942.9	7900.9
AAE@1064-355 nm	1.17	1.53	1.14	1.49	1.1	1.3

At 1064 nm wavelength the optical absorption is not affected by the organic carbon constituents^8^. It is exclusively driven by the mass and the specific absorption cross section of the inorganic carbon—or in optical terminology black carbon (BC)—fraction. Therefore, for the investigation of BC content of the DPM, the optical absorption measured at 1064 nm wavelength is used. Towards the shorter wavelengths, the absorption of the organic carbonaceous particulate matter is gradually increasing and become dominant in the UV wavelength domain i.e. 355 nm. The measured optical absorption clearly fits to this trend in the 1064 nm- 355 nm wavelength range. However, the optical absorption measured at 266 nm appears out of alignment from the fitting with no obvious tendency. The expected values of OAC measured at 266 nm wavelength can be smaller or larger than the fitting values without any identifiable correlation with the operational parameters of the engine or with fuel types. One possible explanation of this irregular behaviour can be speculatively explained by the gas phase’s effect on the measured absorption or other physicochemical phenomena which may take place simultaneously in the complicated vapor-particle transformation processes in a highly reactive and turbulent tailpipe ambience. However, the investigation of this phenomenon is far beyond the scope of this study. Therefore, here we only report the measured results of 266 nm optical absorption (Table 5), but this wavelength is not used for the data analysis throughout this work.

For the investigation of Organic Carbon (OC) or in optical terminology Brown Carbon (BrC) contribution to a total optical absorption, 355 nm data and the wavelength dependency of optical absorption quantified by AAE are used in this study.

At reference temperature (40 °C) the emission at idle speed (em#1) provides the smallest optical absorption value at any operational wavelengths of the 4λ-PAS regardless of the applied fuel. With increasing engine load, the emitted particulates show increasing trends in the measured optical absorption at all wavelengths independently of the applied fuels (Fig. 3). The gradually increasing tendency towards the higher loads in the measured absorption at 1064 nm means that the inorganic BC content of emission is increased inside the emitted particle assembly. The B7 fuel provides smaller optical absorption at 1064 nm at any set of operational conditions of the engine than B0 fuel. So, in comparison, one can conclude that the B7 fuel emitted a smaller amount of BC than B0 fuel at any engine mode. At reference temperature, the optical absorption measured at 355 nm shows the same tendency but as it is expected, it shows remarkably higher absorption values than those measured at 1064 nm (Fig. 3). Increasing the temperature, the measured absorption at 1064 nm shows increasing deviation at a given engine mode towards the higher loads relative to that measured at the reference temperature regardless of the fuel types (Table 5). At higher temperatures, the volatile organic fraction of the aerosol transforms from particle to gas phase through evaporation. Therefore, the small differences in OAC measured at (em#1) and (em#2) at different temperature means that the measured carbonaceous particulate matter includes small amounts of organic volatile fraction, which might evaporate even at 300 °C at 1064 nm. At (em#3) the deviation of the measured absorption is higher than those of (em#1) and (em#2) (Fig. 4). At 355 nm, the tendency is the same but with remarkably higher deviations at any engine mode and fuel types (Fig. 4 and Table 5). This behaviour is well explained by the fact that towards the shorter wavelengths the absorption of the organic carbon content is gradually increased. The higher optical absorption of B7 fuel can also be interpreted by assuming higher organic content of FAME blended in B0 fuel than that of pure B0 fuel.

The AAE values deduced from the measured absorption at the operational wavelengths of the multi-wavelength photoacoustic instrument at all measurement conditions are drawn in Fig. 5 and summarised in Table 5.

The measured OAC and AAE data changes with the changes of the engine modes and posterior temperature treatment. However, it is worth to note, that the OAC is an extensive, while the AAE is an intensive (concentration independent) quantities. Therefore, the OAC is dominantly affected by the emission of mass of the measured analyte, while the AAE is dominantly affected by the cumulative absorption of organic and inorganic fraction of the sample as well as the mixing state of it. As regards the wavelengths dependency of the measured optical absorption quantified by the AAE value, the following data interpretation can be made. At reference temperature, the highest AAE value is measured at idle speed (em#1), while towards the higher rpm and load settings, the spectral dependence of absorption is gradually decreased. Using B7 fuel, the same tendency has been observed, however, the values of AAE are remarkably higher at any engine modes. Increasing the temperature from reference up to 150 °C or even 300 °C, the AAE value is decreasing regardless of the applied fuel and operational conditions of the engine. With both fuel types, the highest deviations have been observed at (em#1). The AAE is the only real time measurable physical quantity of the aerosol that relates to chemical composition and has climate relevancy^27^. Moreover, real time measurable AAE quantity can be correlated to the off-line measurable EC/OC ratio, which is also a crucial parameter for DPM characterisation^28^. The correlation between chemical reactiveness and spectral responses is defined and experimentally verified in detail in many earlier studies^7,15,29^. Based on that, an AAE value around 1 means the dominance of the elemental or black carbon fraction having higher EC/OC (BC/BrC) ratio, while higher values of AAE indicate the presence of organic or in spectral terminology BrC fraction in the particle assembly (lower EC/OC ratio)^30–32^. In this context, Fig. 5 can be interpreted as follows: the relatively high AAE value measured at (em#1) indicates the presence of organic carbon with high absorption ability. Increasing the rpm and torque, the AAE value is decreasing which indicates increasing elemental and black carbon dominance in the emitted particle assembly. The lowest AAE value of (em#3) (AAE = 1.2) points to a negligible organic carbon content^33,34^. This concurs with the fact that the incompleteness of combustion in diesel engines is decreased as torque output increases, resulting in less organic carbon emission. Applying B7 fuel and increasing the sampling temperature, the same tendency has been observed but with higher AAE values at any given em. This behaviour is simply reflecting the higher amount of organic compounds with low chemical reactiveness in B7 than in B0 fuel. The additional decreasing pattern of AAE for B7 at higher temperature can be explained by the fact that chemical composition has changed at higher temperature, since the volatile organic content has evaporated. The measured OAC’s values of diesel soot is increasing with substantial decrement in the number concentration from em#1 to em#2 transition which also implies that chemical composition of the particles is also changing during this transition. Although the number concentration is nearly the same from em#2 to em#3 transition, the OAC’s still increases, which further indicates that the strengths of carbon content is modified during this transition too. It negates the general perception that lower number concentration corresponds to favourable emission characteristics.

The interpretation of the measured absorption coefficients and AAE values in the context of number concentration and size distribution is also in the focus of this study. Comparing Figs. 2 and 4, some straightforward conclusions can be made. The TNC values decreased than remained constant in em#1 to em#2 and em#2 to em#3 transitions subsequently, while the OAC values are decreasing but with different rates towards the higher rpm and torque output. The Photoacoustic (PA) response is exclusively sensitive for carbonaceous particulate matter including BC and BrC (elemental or organic carbon) content, which means that the smaller number-concentration includes a higher carbon content in em#2 than in em#1. In a similar manner, despites of the more or less comparable amount of emitted particles, higher carbon content was measured in (em#3) than in (em#2). Since the PA response is proportional with the mass concentration and absorption ability of the measured aerosol, it is more practical to interpret the spectral responses in the context of TVC values. In this context, at the (em#1) to (em#2) transition the measured TVC values remained more or less constant, while the optical absorption showed increasing tendency. As in the (em#2) to (em#3) transition the TVC and the OAC values show a similarly increasing tendency but with a different rate. It also means that changing in mass concentration does not necessarily follow the changes in strengths of different carbon content in emission. Since the AAE is an intensive quantity (does not depend on the mass-concentration of the measured sample), it provides powerful tool to qualitatively investigate the strengths of different carbon content in emission.

One of the key advantages of the proposed approach is that, using the OAC measurements by the instrument combination 4λ-PAS, TD and SMPS, we cannot just only describe the spectral response but also take another view on the size distribution using spectral response in the context of its thermal stability.

## Conclusions

The primary goal of this work was to demonstrate the applicability of multi-wavelength PA spectroscopy for the qualitative investigation of diesel emissions and thermal particle evolution using a multi wavelength PA instrument-thermodenuder (PA-TD) unit combination. The presented results demonstrate that the parallel measurement of DPM size and spectral responses, that enables the interpretation of size measurement results in a deeper context. The number-concentration and the population statistics including TNC, TVC and GMD values as well as the absorption responses including OAC and AAE data defined at different operational wavelengths were measured at three different engine operating setting using pure petroleum based B0 and B7 fuels. The thermal evolution of the diluted diesel particulates was also investigated at all measurement conditions. The data presented here further confirmed experimentally that diesel emission generally provides lognormal distribution below 100 nm GMD regardless of the fuel type and operational conditions of the engine (Fig. 2). However, we reveal and quantify the bimodal distribution of emission at a particular engine mode. The TNC, TVC, GMD and GSD values were deduced from the measured number-concentrations at a given em using two types of fuel. We experimentally demonstrated a retrograde tendency of TNC and TVC values explained by the dimension scaling and different population statistics of emitted particles (Fig. 2 and Table 4). For the investigation of spectral dependency of DPM quantified by AAE, we used a multi-wavelength PA method. The optical absorption coefficient and the AAE values of emission were measured at the operational wavelengths of the multi-wavelength PA instrument (Fig. 4). The absorption spectra quantified by its wavelength dependency (AAE) were deduced from the measured data at the operational conditions of the engine using different type of fuels (Fig. 5 and Table 4). We observed increasing tendency of OAC and decreasing tendency of AAE values towards the higher rpm and torque settings regardless of the applied fuels (Figs. 4 and 5). Finally, thermal evolution of the investigated emission was also discussed in detail.

The measurement of the spectral responses of temperature treated DPM promises a powerful method for deeper understanding of the environmental impact of DPM. Take advantage of the favourable attributes of PA-TD technique for real-time, precise and accurate measurement of light absorption by DPM aerosol opens up novel possibilities for the investigation of volatility and thermal evolution of diesel emissions and efficiently supports the emission based eco-friendly fuel development as well.

## Data Availability

The datasets used and analysed during the current study available from the corresponding author on reasonable request.
